# Supplementary material for: RNA recognition by Npl3p reveals U2 snRNA-binding compatible with a chaperone role during splicing
Source: Nat Commun. 2023 Nov 7;14:7166. doi: 10.1038/s41467-023-42962-4 (PMC10630445; doi:10.1038/s41467-023-42962-4)
Supplement: Supplementary file 1 — Supplementary Information [file 41467_2023_42962_MOESM1_ESM.pdf]

# Supplementary information

**RNA recognition by Npl3p reveals U2 snRNA-binding compatible with a chaperone role during splicing**

Ahmed Moursy[1,4#], Antoine Cléry[1#*], Stefan Gerhardy[1,2,5], Katharina M. Betz[3], Sanjana Rao[2], Jarosław Mazur[2], Sébastien Campagne[1,7], Irene Beusch[1,8], Malgorzata M. Duszczyk[1], Mark D. Robinson[3], Vikram Govind Panse[2,6*], Frédéric H.-T. Allain[1*]

[1] Department of Biology, Institute of Biochemistry, ETH Zurich, Switzerland

[2] Institute of Medical Microbiology, University of Zurich, Switzerland

[3] Institute of Molecular Life Sciences, University of Zurich, Switzerland

[4] current address: Novartis Institutes for BioMedical Research, Basel, Switzerland

[5] current address: Sardona Therapeutics, San Francisco, USA

[6] Faculty of Science, University of Zurich, Switzerland

[7] ARNA laboratory, INSERM U1212, University of Bordeaux, France

[8] Department of Biochemistry and Biophysics, University of California, San Francisco, San Francisco, CA, USA

* Correspondence: allain@bc.biol.ethz.ch or vpanse@imm.uzh.ch or aclery@bc.biol.ethz.ch

# These authors participated equally to this work

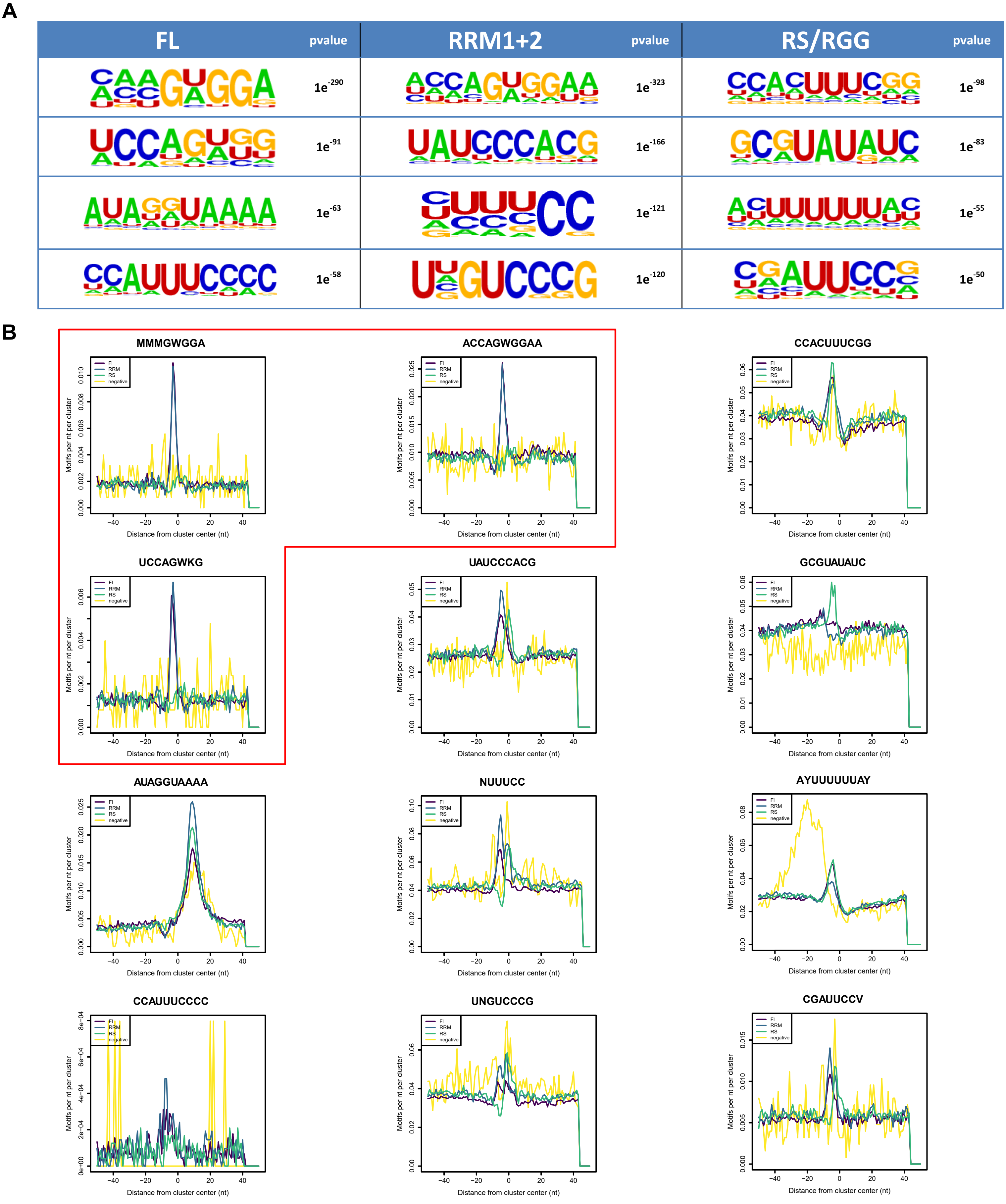


**Supplementary figure 1:** Top enriched motifs of the HOMER de novo motif search for the split-iCRAC. **(A)** The top 4 enriched HOMER motifs in the crosslink clusters ±5 nt for the different constructs of Npl3 compared to the random background. The p-value of each motif indicates its enrichment in the corresponding sample over random sequences of similar length from the S. cerevisiae genome. **(B)** Density plot for each of the enriched motifs in the 100 nucleotides around the crosslinking cluster centers in the different samples and the negative control. Traces for the full-length protein, RRM1/2, RS and negative control are colored in purple, blue, green and yellow, respectively. All motifs show similar densities in the different samples compared to the negative control except the three motifs boxed in red. Those three motifs have significantly higher densities immediately preceding the crosslinking cluster center in the FL and RRM1/2 samples.

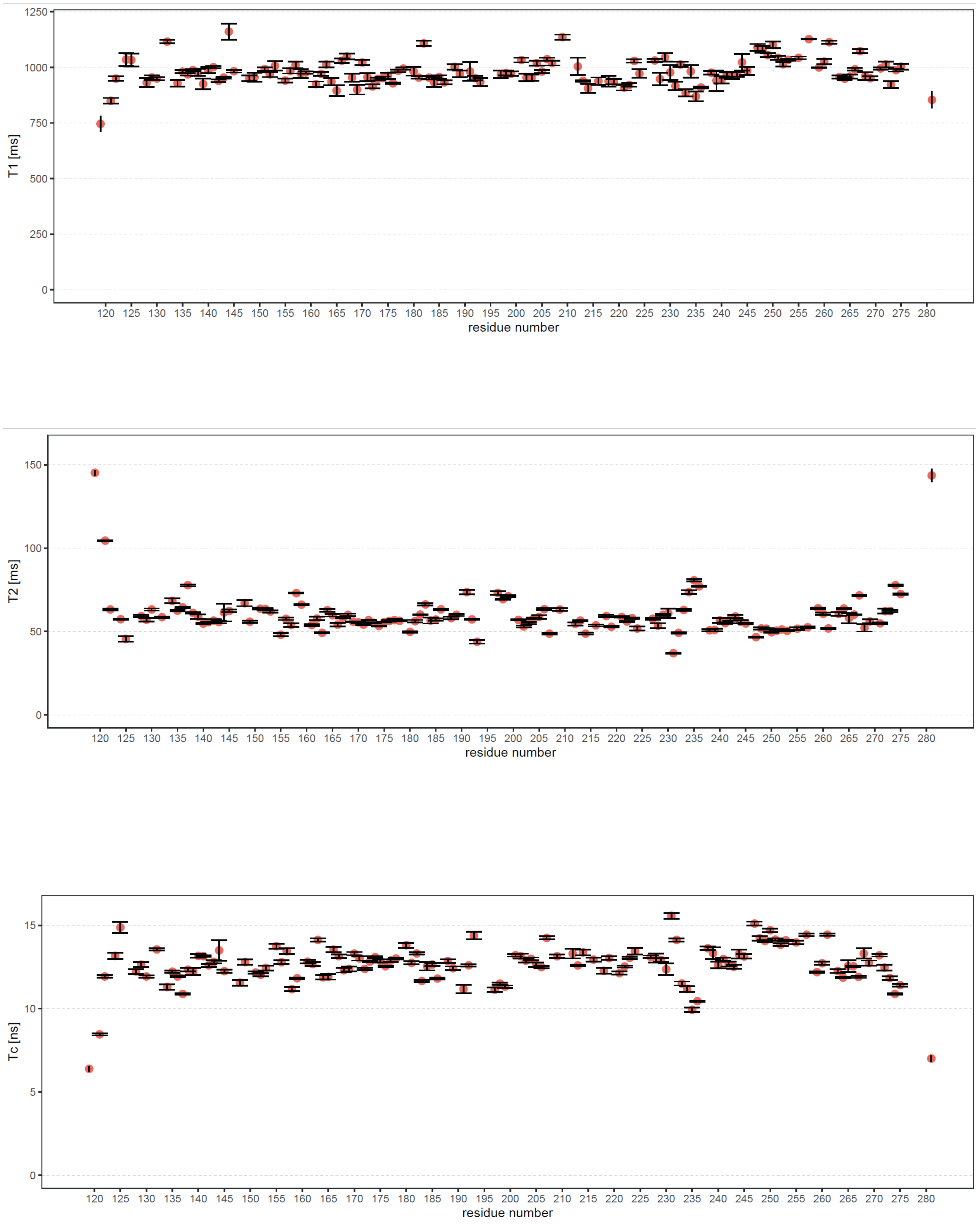


**Supplementary figure 2:** Representation of the T1, T2 and correlation time (τc) measured in the presence of Npl3 RRM1/2 bound to the AUCCAGUGGAA RNA. Mean values and standard deviations are shown. The two RRMs tumbled with RNA as a single unit. Source data are provided as a Source Data file.

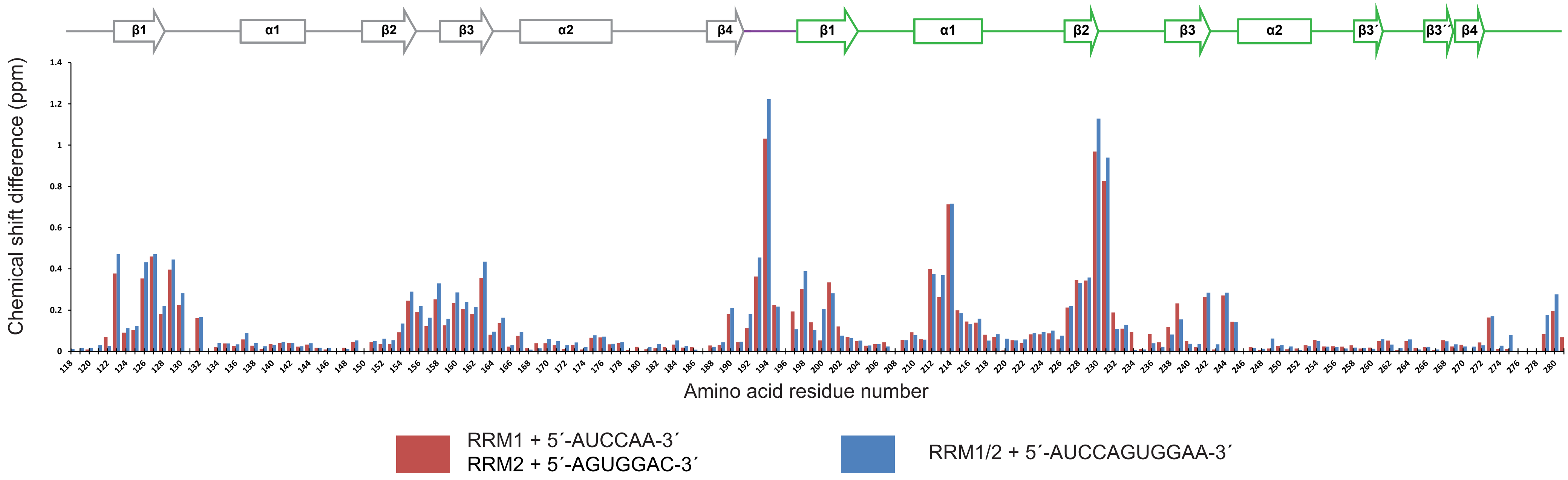


**Supplementary figure 3:** Comparison of the binding of Npl3 RRM1/2 to the 5´-AUCCAGUGGAA-3´ RNA with isolated RRM complexes. Representation of the combined chemical shift perturbations of Npl3 RRM1/2 amide residues upon binding to the 5´-AUCCAGUGGAA-3´ RNA (in blue) at a ratio of 1:1 in comparison with the isolated RRM domains bound to their respective RNA targets (in red). The corresponding secondary structure elements are represented at the top of the graph. RRM1, RRM2 and the inter-domain linker are colored in gray, green and magenta, respectively. Some residues could not be assigned in the bound form of RRM1/2 due to the intermediate to slow exchange regime. Source data are provided as a Source Data file.

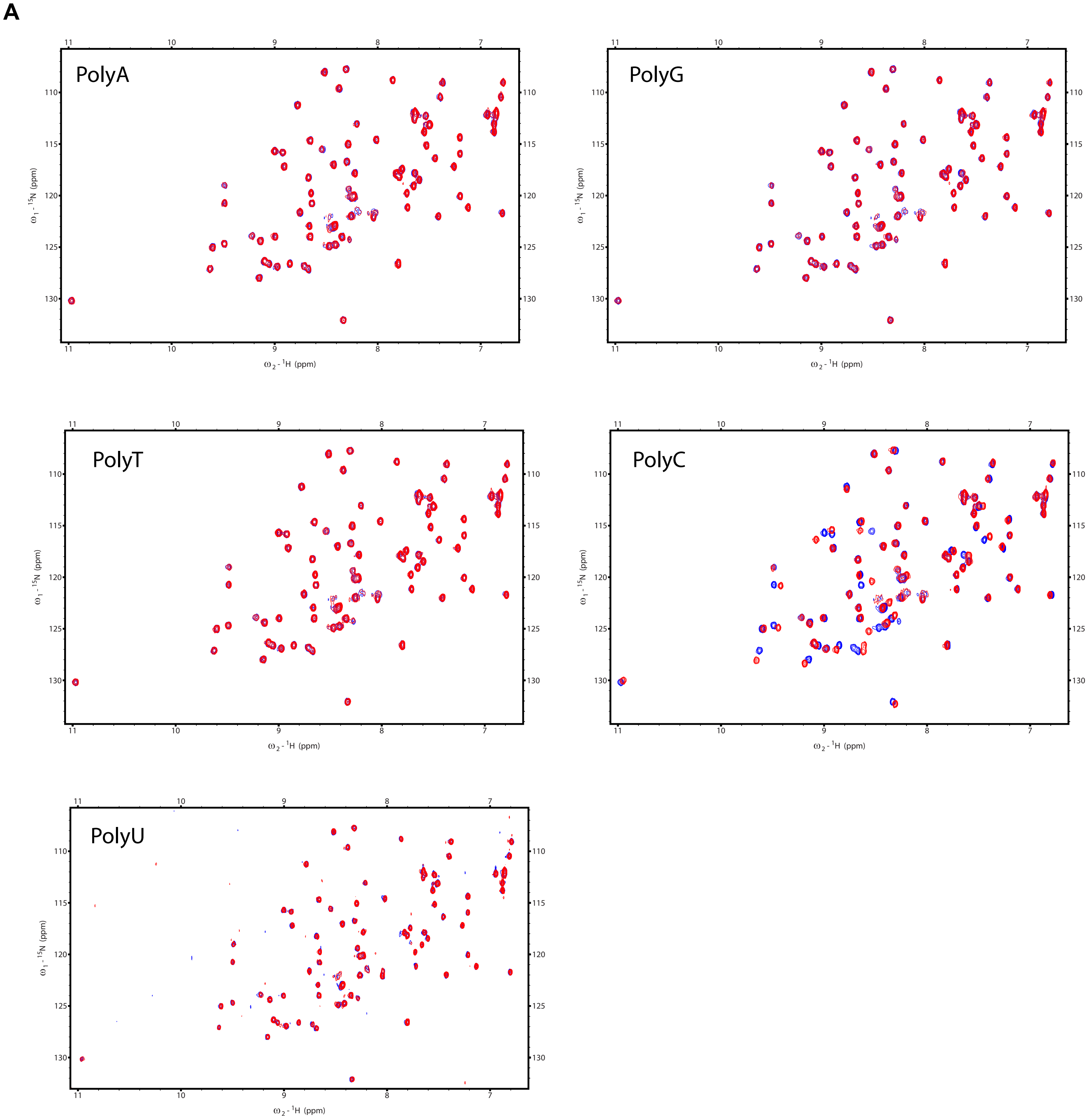
A
PolyA
PolyG
PolyT
PolyC
PolyU
ω1 - 15N (ppm)
ω2 - 1H (ppm)
11
10
9
8
7
110
115
120
125
130

**B**

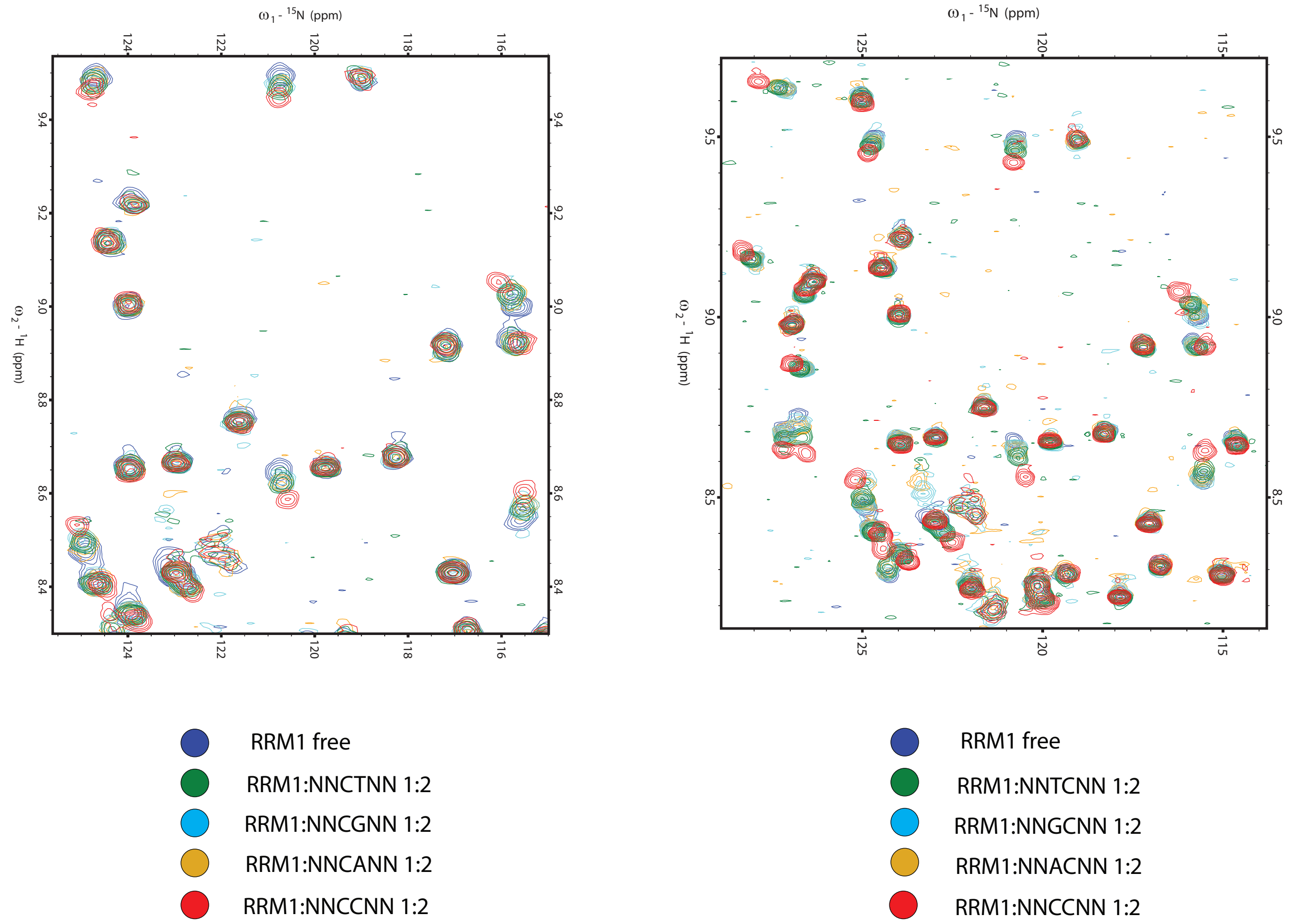


**Supplementary figure 4:** Npl3 RRM1 binds preferentially to polyC sequences. **(A)** Overlay of 1H-15N HSQC spectra recorded with Npl3 RRM1 upon titration with 6mer polyA, polyG, polyT and polyC ssDNA as well as 8mer polyU RNA sequences. The spectra were recorded at 30°C, in the Npl3 RRM1/2 NMR buffer. Blue represents the free protein while red represents the bound protein at a 1:1 ratio. **(B)** Overlay of 1H-15N HSQC spectra recorded with Npl3 RRM1 upon titration with NNCxNN DNA (left panel) and NNxCNN DNA (right panel); x is for A, C, G or T. The free protein is in blue, the bound form at a 1:2 ratio in protein:DNA is in green for NNCTNN or NNTCNN, cyan for NNCGNN or NNGCNN, orange for NNCANN or NNACNN and red for NNCCNN.

A

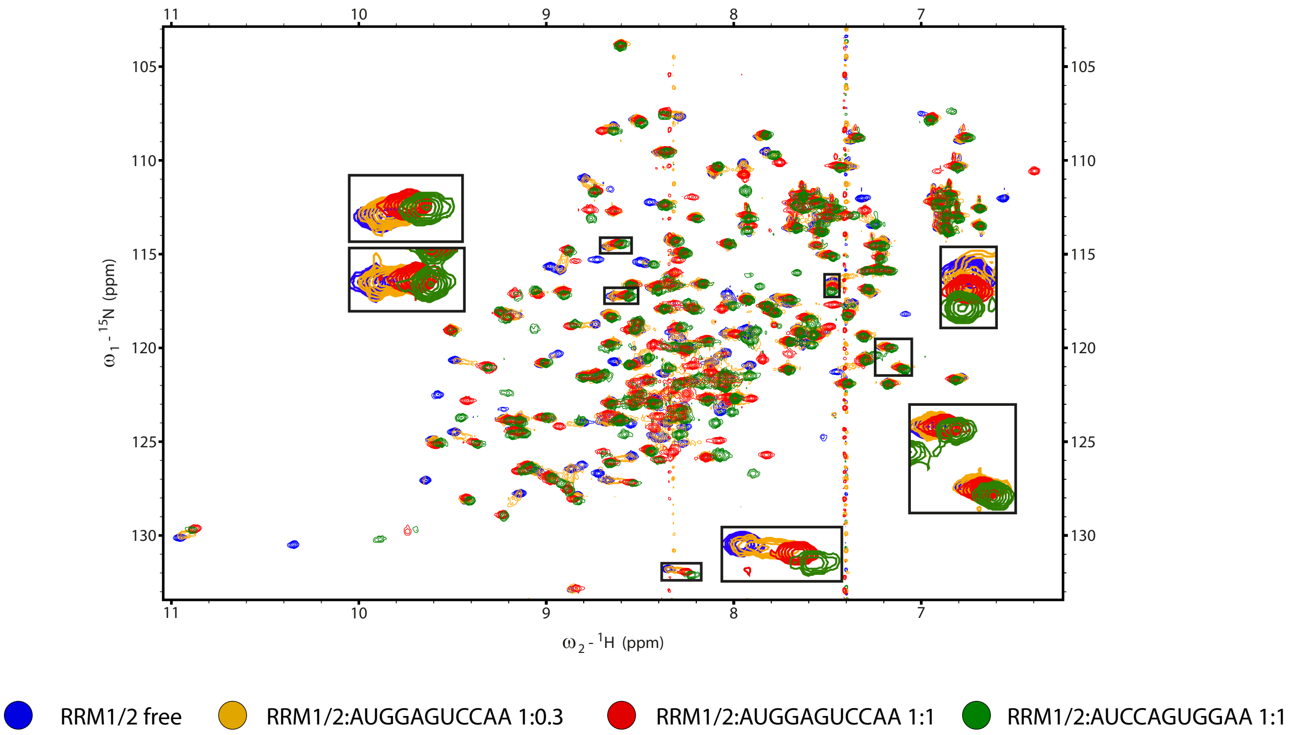


B

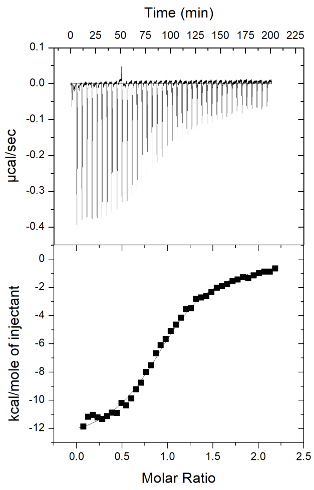


Kd = 1.2 µM, N=1

**Supplementary figure 5:** Study of the interaction of Npl3 RRM1/2 with an RNA containing inverted RRM binding sites (AUGGAGUCCAA). **(A)** NMR overlay of 1H-15N HSQC spectra recorded with Npl3 RRM1/2 free form, bound to AUGGAGUCCAA at 1:0.3 and 1:1 ratios and to AUCCAGUGGAA at a 1:1 ratio. **(B)** ITC measurement performed with Npl3 RRM1/2 and the AUGGAGUCCAA RNA.

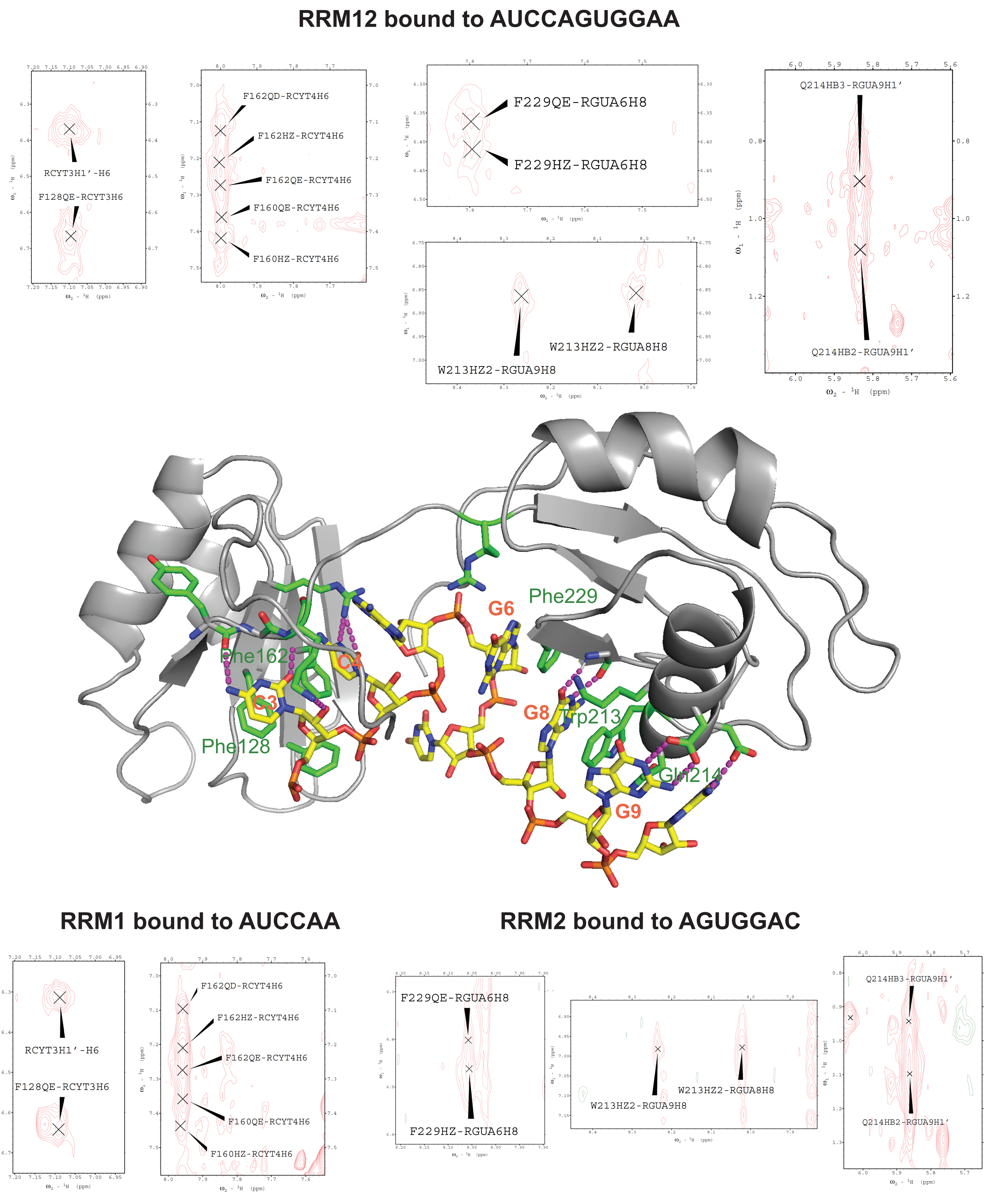


**Supplementary figure 6:** The binding of the two RRMs of Npl3 to RNA is the same when the domains are in isolation or in the context of RRM1/2. Some characteristic inter-NOEs are shown as snapshots of filtered-edited 2D Noesy spectra recorded with RRM1/2, RRM1 or RRM2 bound to RNA. The same pattern of inter-NOEs is observed when RRMs are in isolation (RRM1 or RRM2) or linked by their inter-domain linker (RRM1/2).

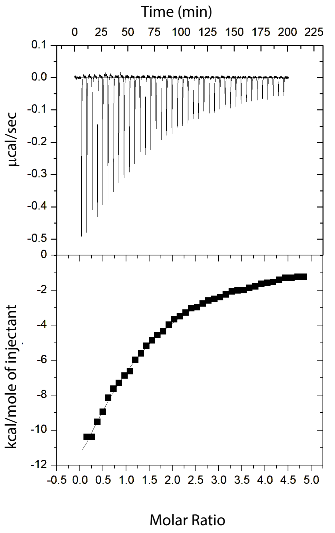


RRM1/2 R126A + AUCCAGUGGAA
Kd = 10.7 +/- 2.3 µM, N=1.1

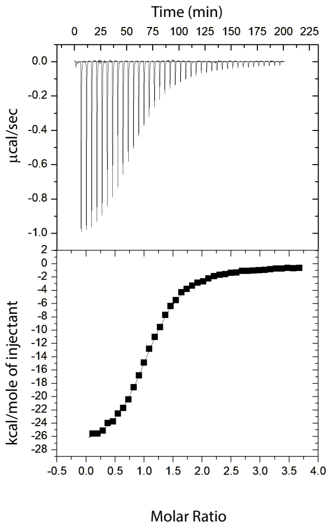


RRM1/2 R130A + AUCCAGUGGAA
Kd = 0.9 +/- 0.3 µM, N=1.1

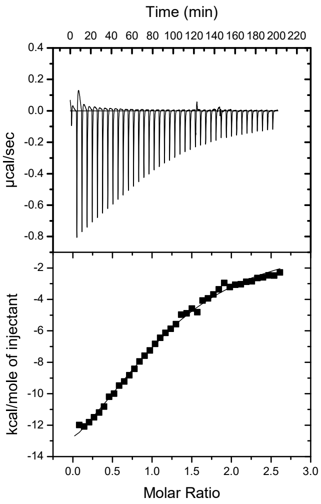


RRM1/2 F128A + AUCCAGUGGAA
Kd = 12.8 +/- 0.8 µM, N=1.1

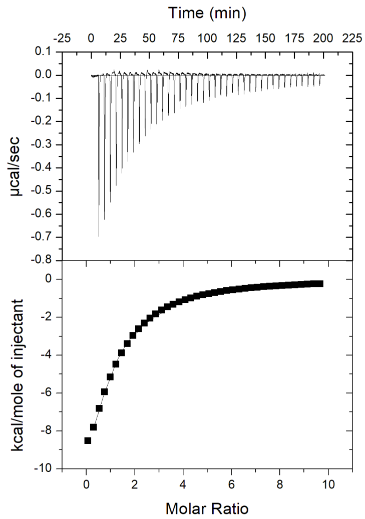


RRM1/2 F128AF160A + AUCCAGUGGAA
Kd = 15.6 +/- 3.6 µM, N=0.9

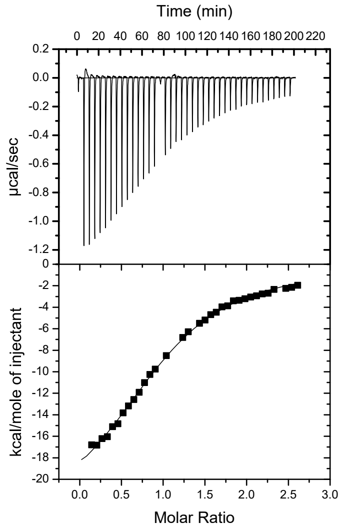


RRM1/2 K217A + AUCCAGUGGAA
Kd = 7.5 +/- 0.5 µM, N=1

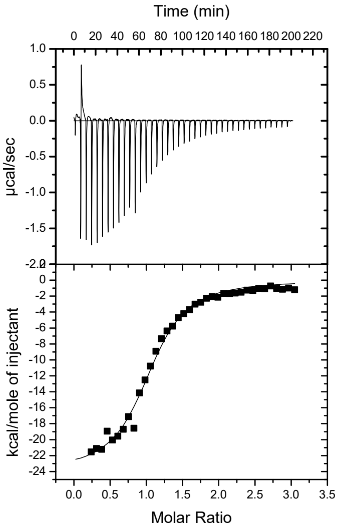


RRM1/2 Q214A + AUCCAGUGGAA
Kd =1.7 +/- 0.2 µM, N=1.1

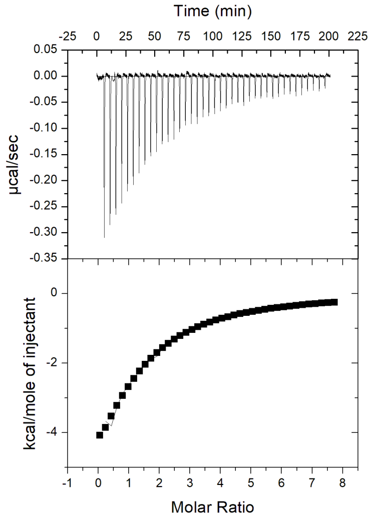


RRM1/2 Q214AK217A + AUCCAGUGGAA
Kd =15.3 +/- 0.6 µM, N=1.1

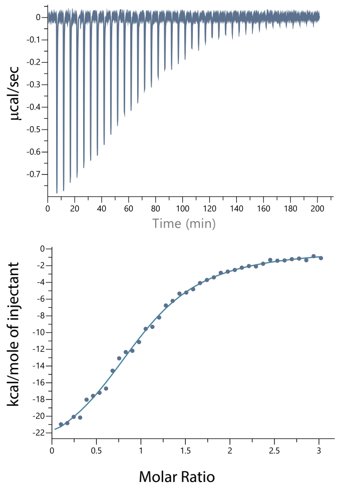


RRM1/2 F229A + AUCCAGUGGAA
Kd = 2.3 +/- 0.3 µM, N=1

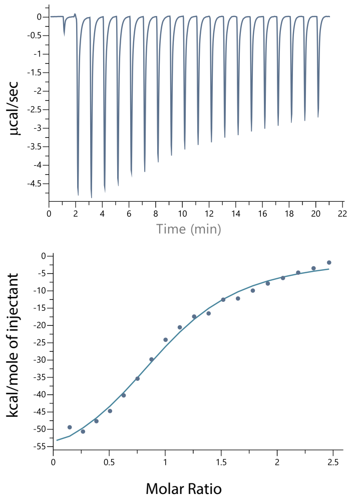


RRM1/2 LPA->GGG + AUCCAGUGGAA
Kd = 3.8 +/- 0.2 µM, N=1

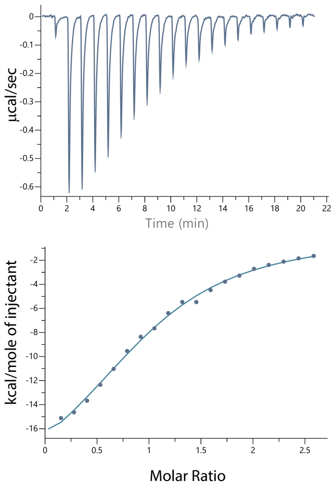


RRM1/2 LPA->$(GGGGS)_3$ + AUCCAGUGGAA
Kd = 6.8 +/- 0.9 µM, N=1

**Supplementary figure 7:** ITC measurements performed with the AUCCAGUGGAA RNA and different variants of the Npl3 RRM1/2 protein. All measurements were done in duplicates.

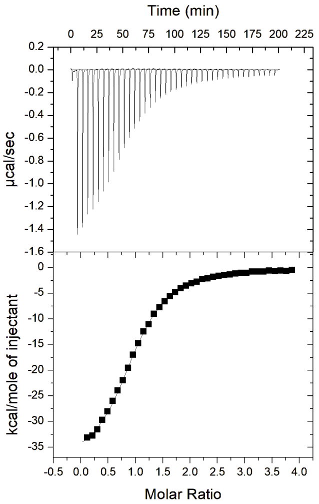


RRM1/2 + AUUCAGUGGAA
Kd = 1.8 +/- 0.5 μM, N=1

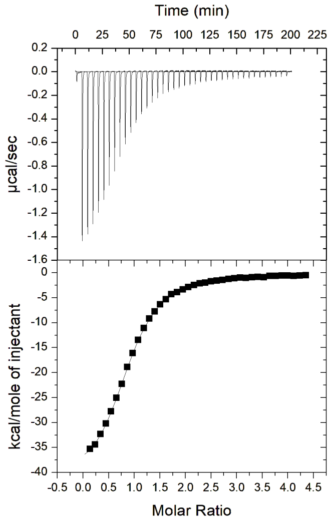


RRM1/2 + AUCUAGUGGAA
Kd = 1.6 +/- 0.4 μM, N=1

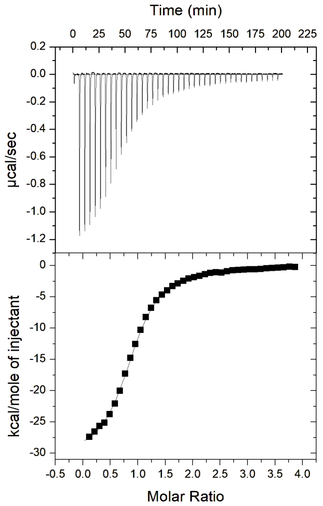


RRM1/2 + AUCCUGUGGAA
Kd = 1.3 +/- 0.5 μM, N=0.9

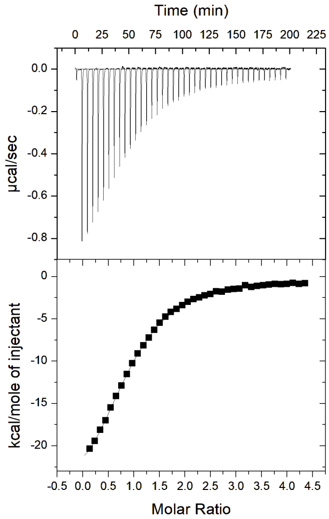


RRM1/2 + AUCCAAUGGAA
Kd = 2.2 +/- 0.5 μM, N=0.9

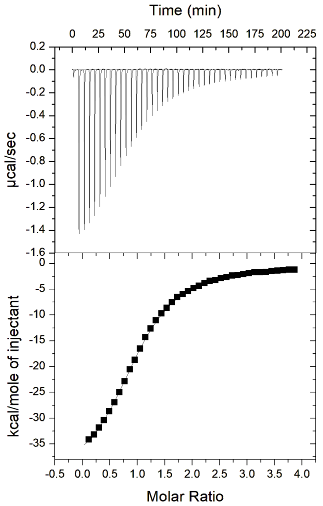


RRM1/2 + AUCCAGUAGAA
Kd = 1.9 +/- 0.1 μM, N=1

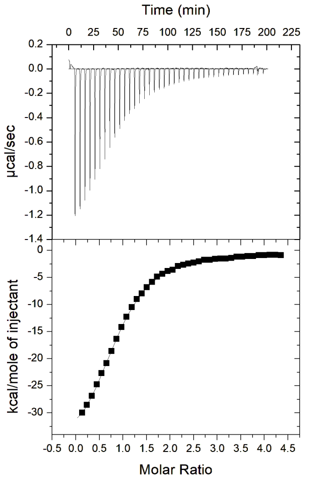


RRM1/2 + AUCCAGUGAAA
Kd = 2.5 +/- 0.5 μM, N=0.9

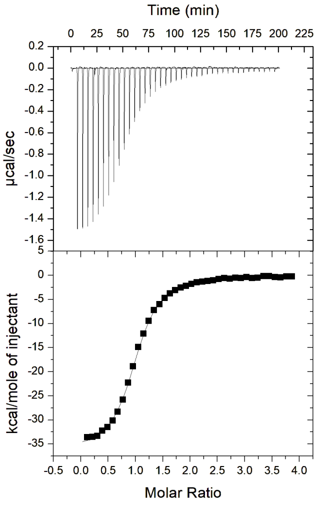


RRM1/2 + AUCC-GUGGAA
Kd = 0.9 +/- 0.4 μM, N=1

**Supplementary figure 8:** ITC measurements performed with Npl3 RRM1/2 and different variants of the AUCCAGUGGAA RNA. All measurements were done in duplicates.

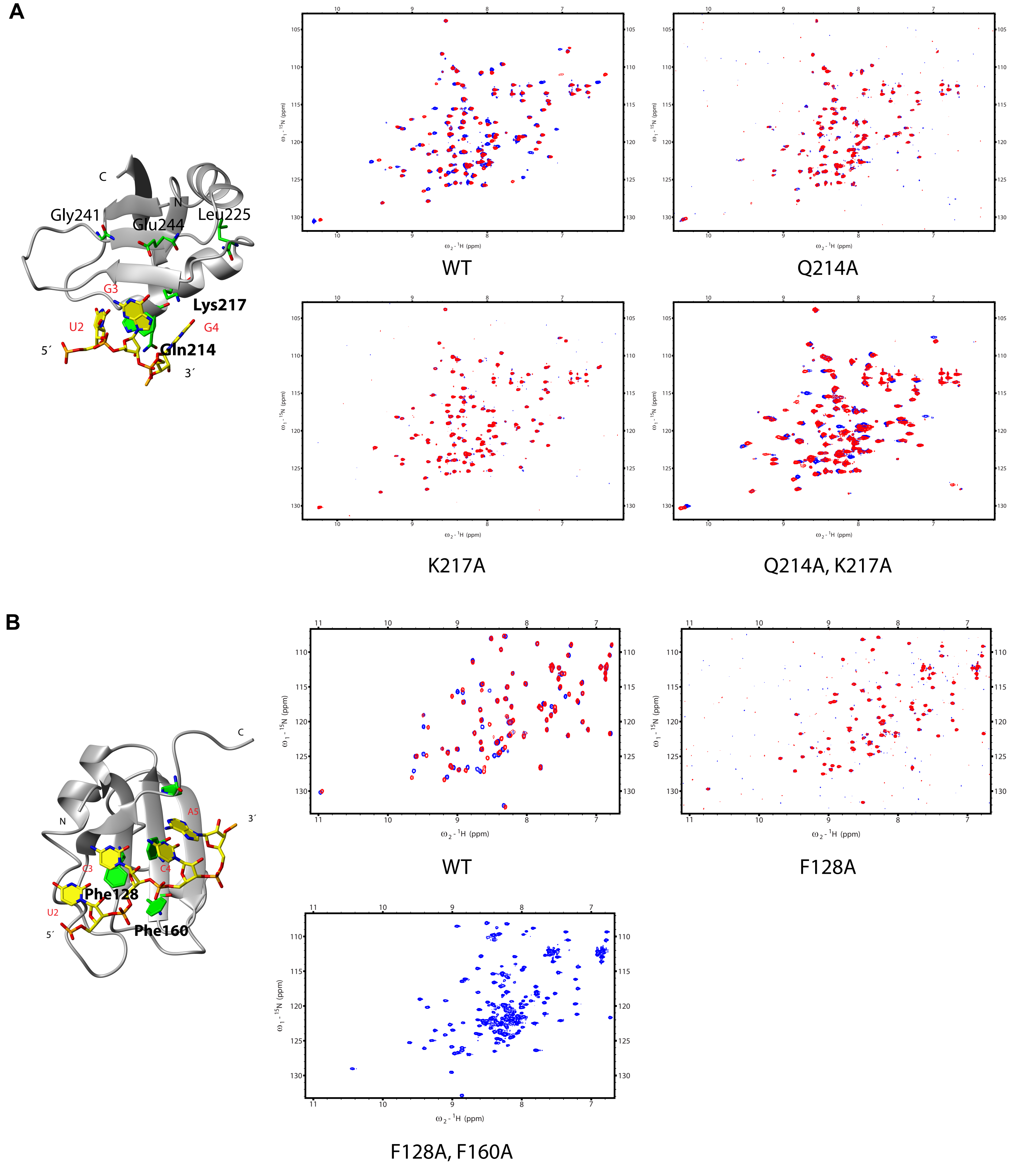


**Supplementary figure 9:** Point mutations in RRM1 and RRM2 disrupt their binding to nucleic acid without affecting the fold of the domains. **(A)** Overlay of 1H-15N HSQC spectra recorded with RRM2 mutants in their free form and upon addition of their target sequence at a 1:1 ratio represented in blue and red, respectively. The spectra were recorded at 40°C, in the RRM2 NMR buffer. Binding was tested to 5´-ATGGTC-3´ ssDNA except for the Q214A, K217A double mutant which was done with 5´-AUGGUC-3´ RNA. The mutated residues are highlighted on the structure of the RRM2-RNA complex. **(B)** Overlay of 1H-15N HSQC spectra recorded with RRM1 mutants in their free form and upon addition of their target sequence at a 1:1 ratio represented in blue and red, respectively. The spectra were recorded at 40°C, in the RRM1 NMR buffer. Binding was tested to 6mer polyC ssDNA. The mutated residues are highlighted on the structure of the RRM1-RNA complex. The spectrum of the double mutant F128A, F160A in the bound form was not recorded, but ITC measurement indicates a strong decrease in affinity of this mutant for RNA (Fig. S7).

**A**

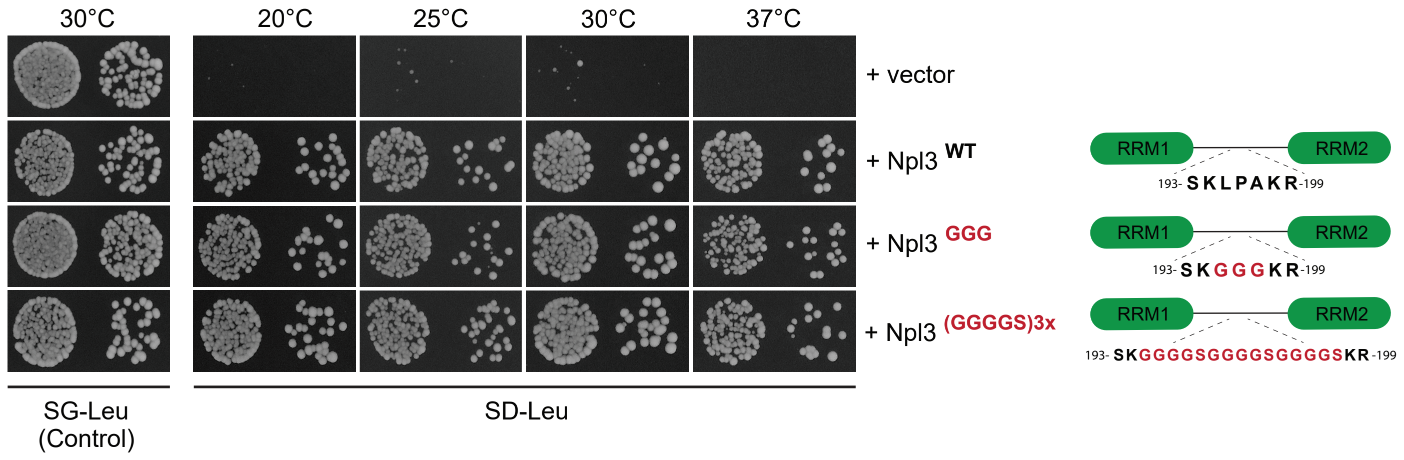
30°C
20°C
25°C
30°C
37°C
+ vector
+ Npl3 WT
+ Npl3 GGG
+ Npl3 (GGGGS)3x
SG-Leu
(Control)
SD-Leu
RRM1
RRM2
193- SKLPAKR-199
193- SKGGGKR-199
193- SKGGGGSGGGGSGGGGSKR-199

**B**

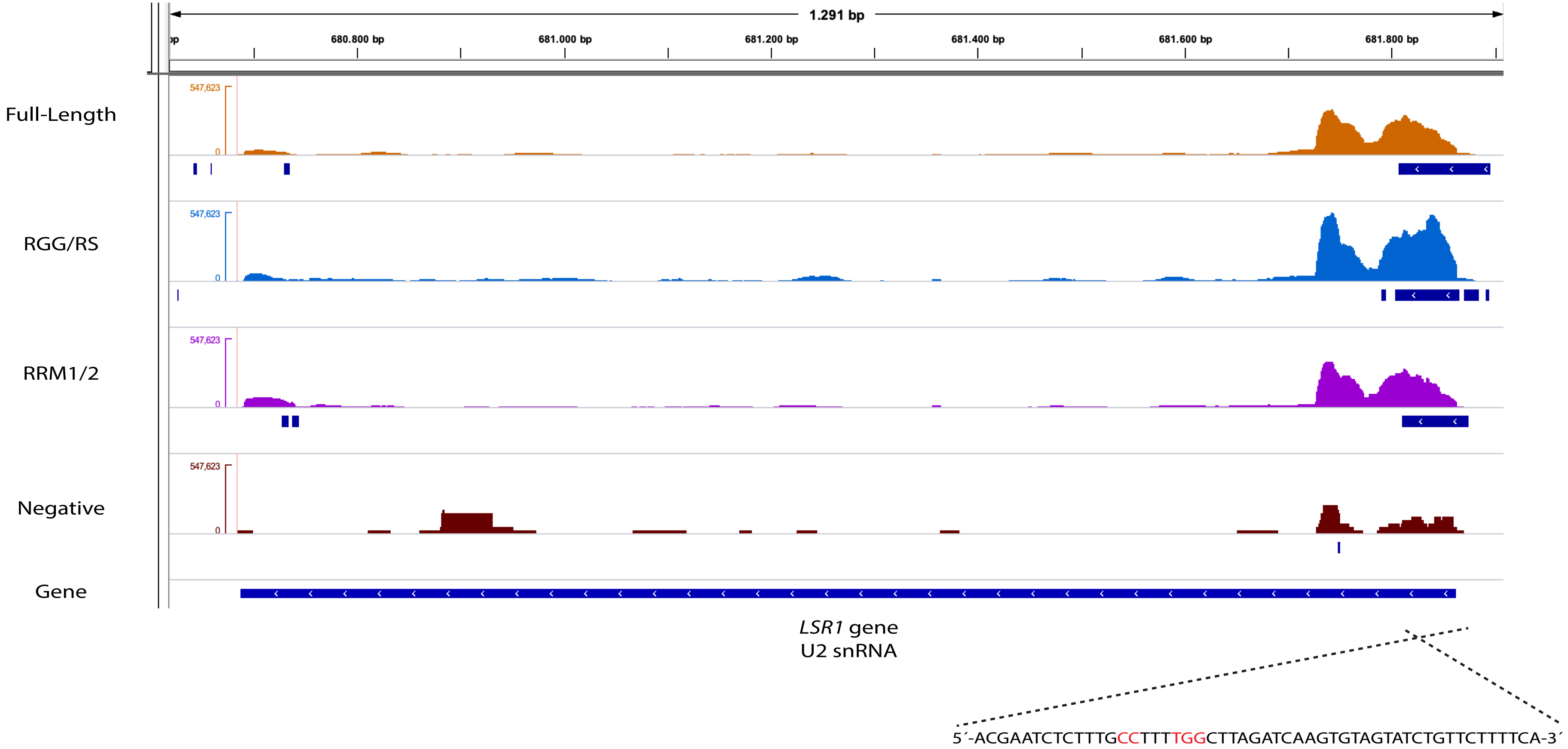
1.291 bp
680.800 bp
681.000 bp
681.200 bp
681.400 bp
681.600 bp
681.800 bp
547,623
0
Full-Length
RGG/RS
RRM1/2
Negative
Gene
*LSR1* gene
U2 snRNA
5´-ACGAATCTCTTTGCCTTTTGGCTTAGATCAAGTGTAGTATCTGTTCTTTTCA-3´

C

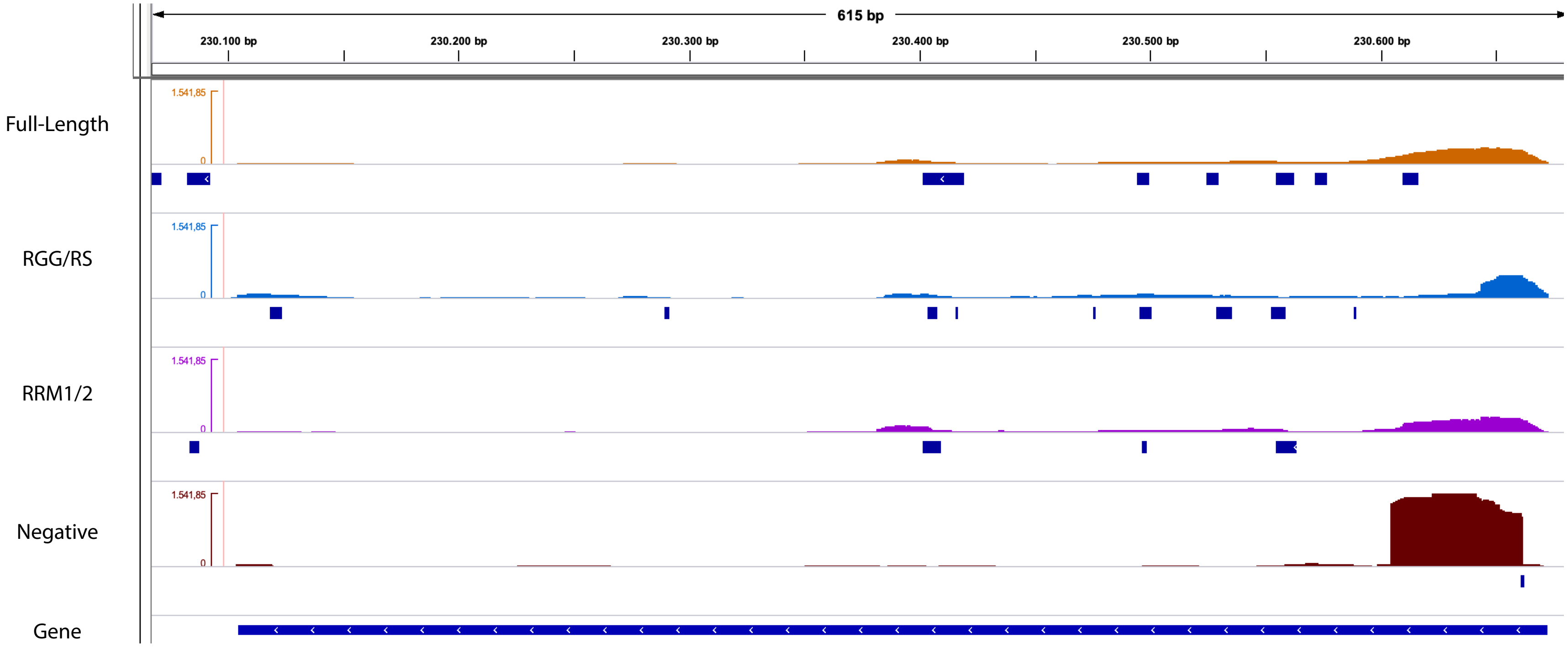


*snR19* gene
U1 snRNA

D

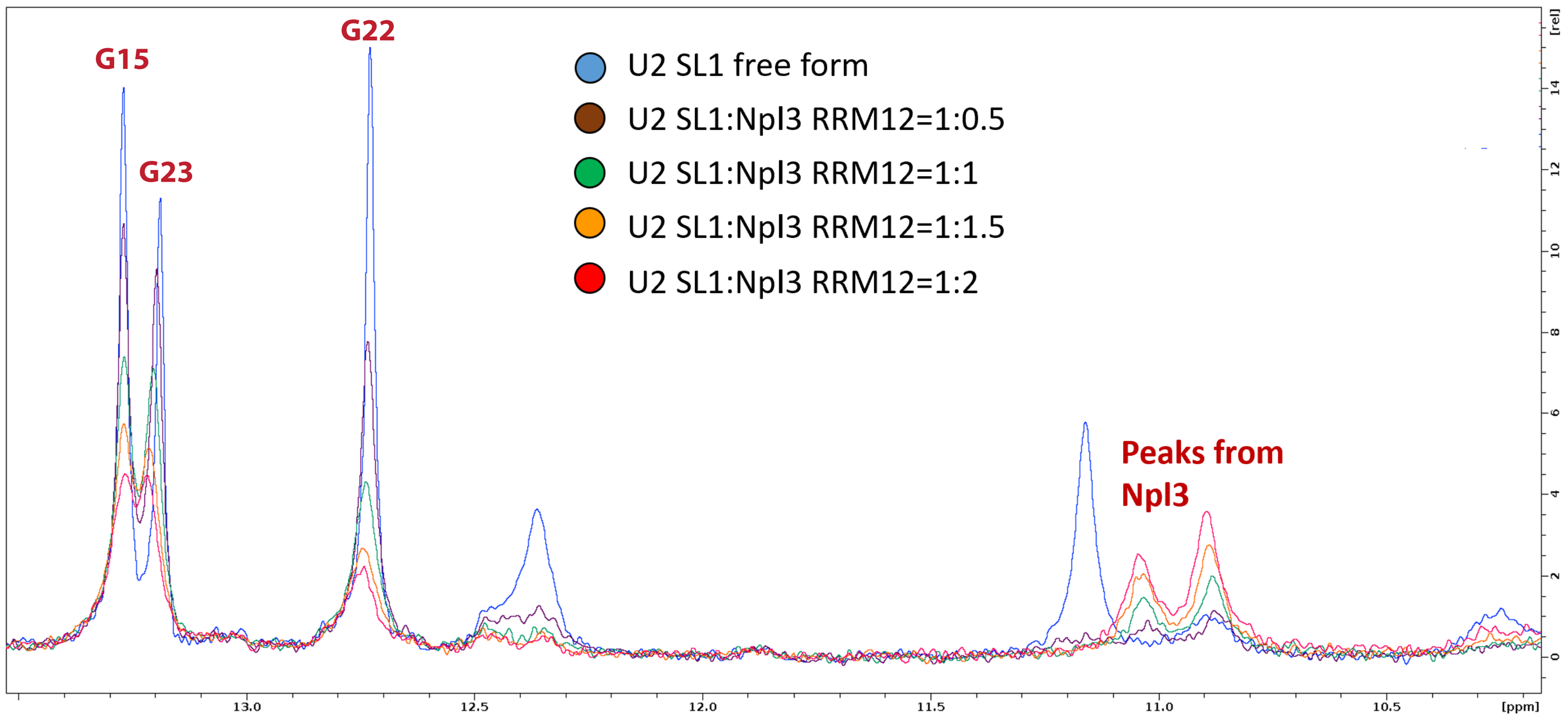
G15
G23
G22
U2 SL1 free form
U2 SL1:Npl3 RRM12=1:0.5
U2 SL1:Npl3 RRM12=1:1
U2 SL1:Npl3 RRM12=1:1.5
U2 SL1:Npl3 RRM12=1:2
Peaks from Npl3
13.0
12.5
12.0
11.5
11.0
10.5
[ppm]
[rel]

E

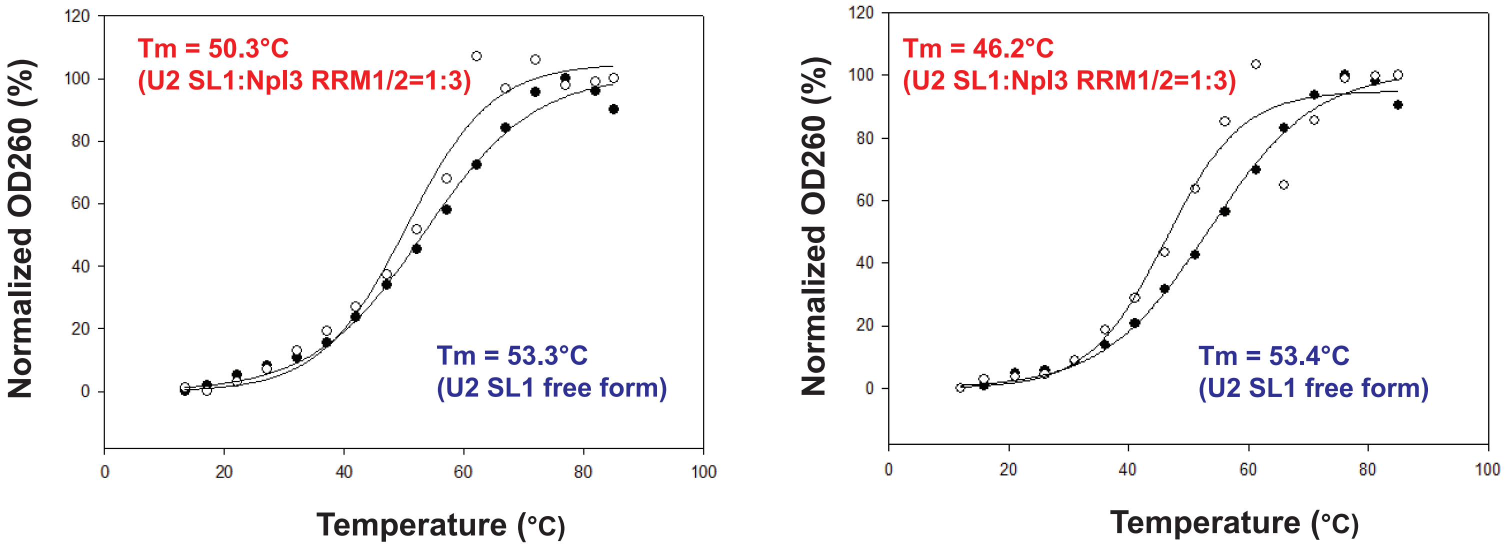


**Supplementary figure 10: (A)** Mutant growth analysis of Npl3Δ strain complemented with vectors expressing different protein variants of Npl3 (marked on the right). Yeast cells were plated on SD-leu plates and incubated either at 20, 25, 30 or 37°C. Two steps of a yeast serial dilution are shown for each condition. The experiment was done in triplicate. **(B)** Direct binding of Npl3 to the U2 snRNA . Genome browser view of the LSR1 gene encoding the U2 snRNA displaying the split-iCRAC coverage tracks of the full-length protein, RGG/RS, RRM1/2 constructs and the negative control. Blue boxes represent the identified crosslinking clusters. The enlarged sequence represents the sequence of the 5´ region of the gene that is represented in the crosslinking clusters of the three protein constructs. **(C)** Similar view as in (B) for the snR19 gene encoding the U1 snRNA showing that no specific crosslinking cluster could be detected with Npl3. **(D)** Overlay of 1D NMR spectra recorded at 303 K with U2 SL I free form and at U2 SL I:Npl3 RRM1/2 ratios of 1:0.5, 1:1, 1:1.5 and 1:2. **(E)** UV melting curve of the U2 SL1 RNA in the free form (black circles) and bound to Npl3 RRM1/2 (white circles). The protein induces a decrease of the RNA melting temperature. The curves were fitted using a sigmoidal equation with 3 parameters: f= a/(1+exp(-(x-xo)/b)). Source data are provided as a Source Data file.

**A**

**B**

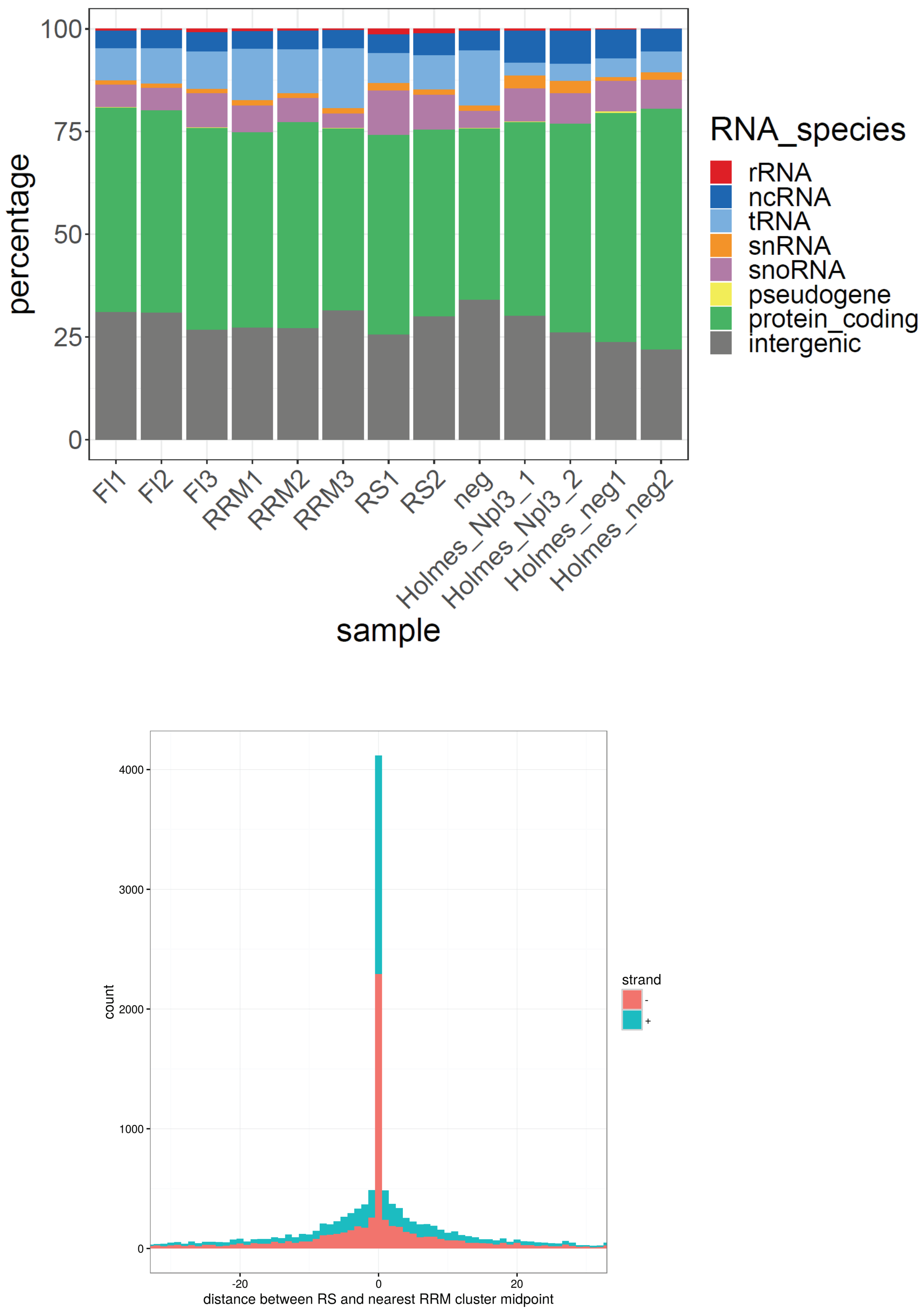


**Supplementary figure 11: (A)** Barplot of the read distribution across different RNA species in the split-iCRAC experiment. Each bar represents one sample and the y-axis the percentage of unique reads mapping to different RNA species as annotated in the Ensembl reference annotation of S. cerevisiae (Saccharomyces_cerevisiae. R64-1-1.83). Data from Holmes et al. (16) were analysed using the same pipeline as for the data obtained in our study for comparison. **(B)** The distance between the RS/RGG domain and the closest RRM cluster midpoint is shown. The RS domain binds RNA on both sites of RRMs crosslinking sites.

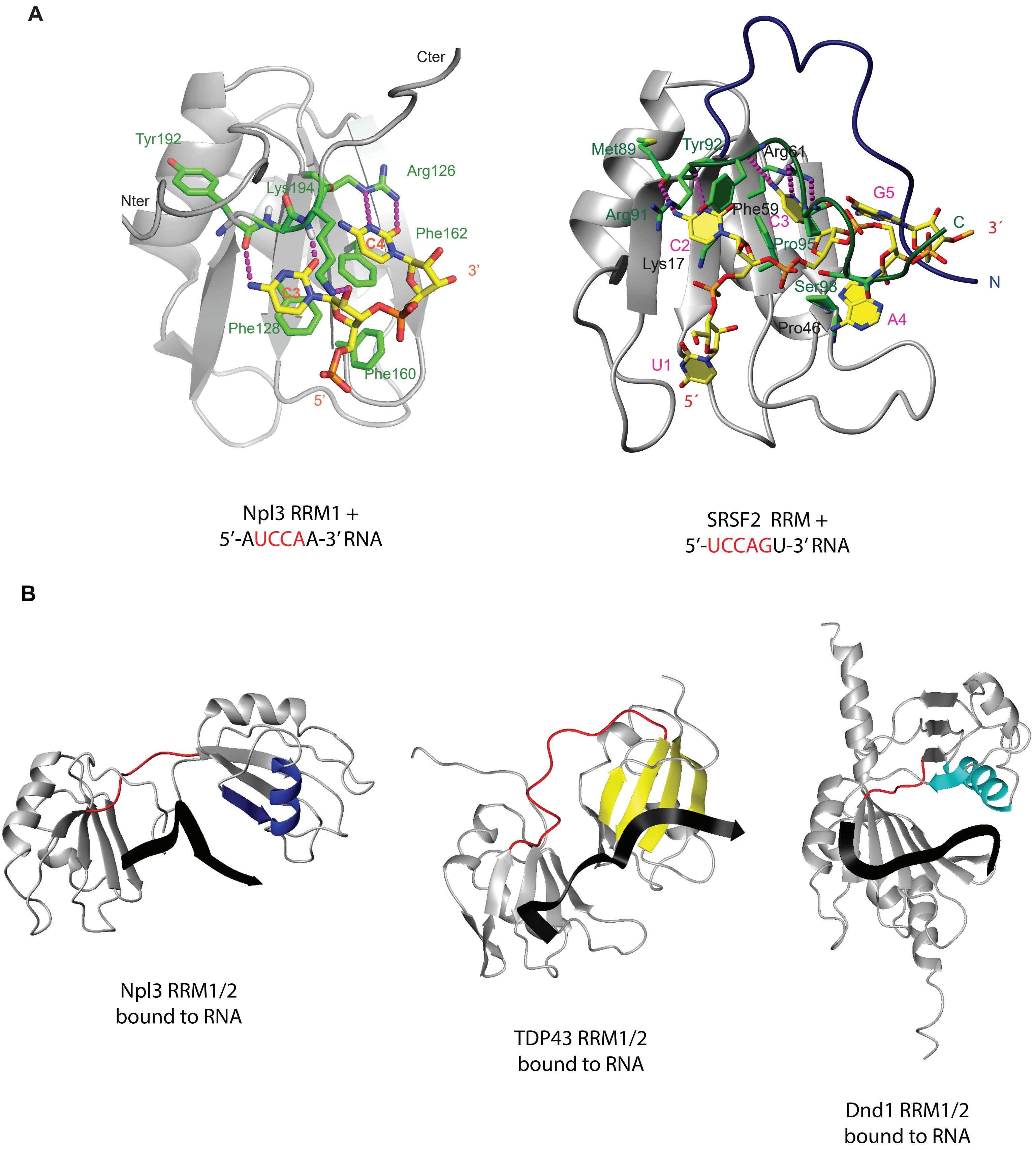


**Supplementary figure 12:** Comparison of the mode of interaction of Npl3 and other RNA binding proteins with RNA. **(A)** Structures of Npl3 RRM1 and SRSF2 RRM bound to RNA. The color code is as in Fig. 4. The two RRMs bind similarly to two consecutive cytosines. **(B)** Schematic representation of the binding interaction of Npl3, TDP43 and Dnd1 RRM1/2 with RNA. The RNA is represented by a black arrow, the inter-domain linker is in red and the part of RRM2 involved in the interaction is colored in blue, yellow and cyan, respectively.

**A**

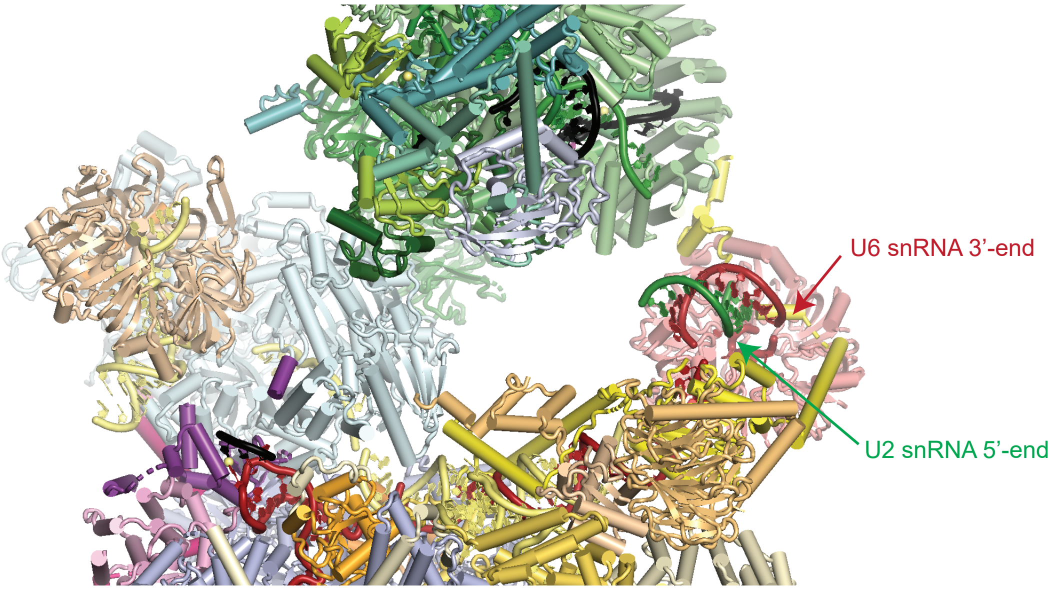


Spliceosome Yeast B complex

**B**

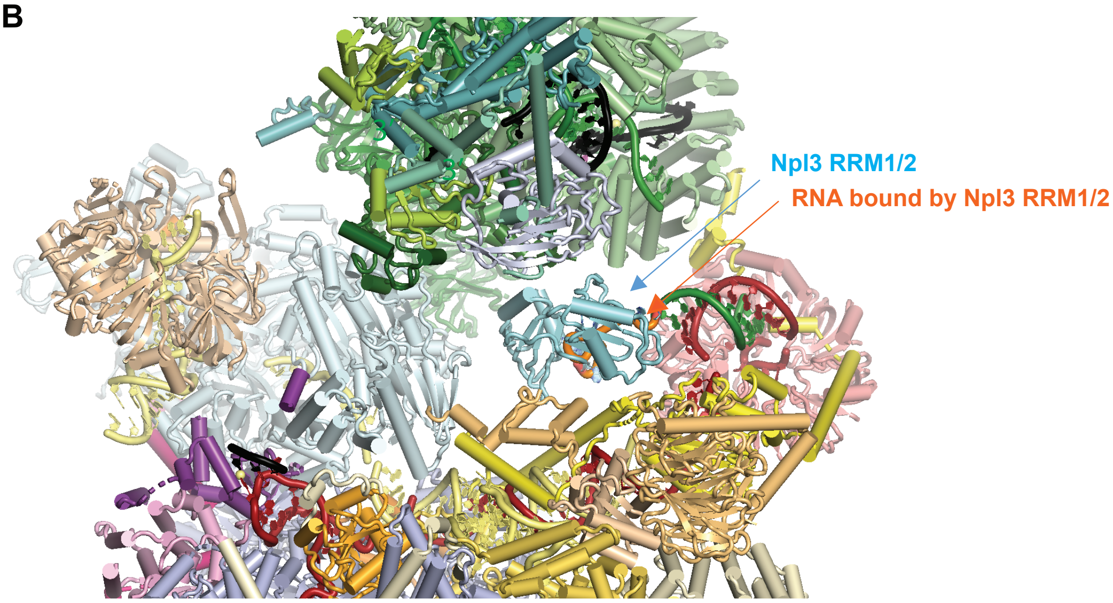


Spliceosome Yeast B complex after positioning Npl3 RRMs bound to RNA on the cryo-EM structure

**Supplementary figure 13:** Npl3 RRM1/2 bound to RNA fits into a cavity present at its U2 snRNA cross-linked site in the yeast spliceosome complex B. **(A)** View of the cryo-EM structure determined with the yeast spliceosome complex B. A large cavity is observed at the position where the Npl3 cross-linked sequence is not visible. **(B)** The structure of Npl3 RRM1/2 bound to RNA fits perfectly in this cavity.

**Supplementary Table 1: Structural statistics of the Npl3 RRM1 in complex with AUCCAA RNA**

| | Protein | | RNA |
|---|---|---|---|
| **NMR distance and dihedral constraints** | | | |
| Distance restraints | | | |
| Total NOE | 2475 | | 58 |
| Intra-residue | 463 | | 40 |
| Inter-residue | | | 18 |
| Sequential ($\|i-j\| = 1$) | 629 | | 0 |
| Nonsequential ($\|i-j\| > 1$ ) | 1359 | | 0 |
| Hydrogen bonds | 24 | | 0 |
| Protein–RNA intermolecular | | 135 | |
| Total dihedral angle restraints | | | 6 |
| Protein | | | |
| $\phi$ | 0 | | |
| $\psi$ | 0 | | |
| Nucleic acid | | | |
| Base pair | | | 0 |
| Sugar pucker | | | 6 |
| Backbone | | | 0 |
| Based on A-form geometry | | | 0 |
| | | | |
| **Structure statistics** | | | |
| Violations (mean and s.d.) | | | |
| Number of distance constraints (>0.3 Å) (Å) | 6.3 ± 2.2 | | |
| Dihedral angle constraints (º) | 0 | | |
| Max. dihedral angle violation (º) | 0 | | |
| Max. distance constraint violation (Å) | 0.43 | | |
| Deviations from idealized geometry | | | |
| Bond lengths (Å) | 0.0042 ± 0.0001 | | |
| Bond angles (º) | 1.228 ± 0.016 | | |
| Average pairwise r.m.s. deviation** (Å) | | | |
| Protein | | | |
| Heavy | 0.41 ± 0.06 | | |
| Backbone | 0.14 ± 0.02 | | |
| RNA | | | |
| All RNA heavy | | | 0.30 ± 0.05 |
| Complex | | | |
| Protein and RNA heavy | | | 0.41 ± 0.06 |

** Protein r.m.s. deviation was calculated using residues 125 to 195 for the ensemble of 10 refined structures. RNA r.m.s. deviation was calculated using nucleotides 110 and 112 for the ensemble of 10 refined structures.

**Supplementary Table 2: Structural statistics of the Npl3 RRM12 in complex with AUCCAGUGGAA RNA**

| | Protein | | RNA |
|---|---|---|---|
| **NMR distance and dihedral constraints** | | | |
| Distance restraints | | | |
| Total NOE | 3788 | | 63 |
| Intra-residue | 728 | | 47 |
| Inter-residue | | | |
| Sequential ($\|i-j\| = 1$) | 1014 | | 16 |
| Nonsequential ($\|i-j\| > 1$ ) | 2008 | | 0 |
| Hydrogen bonds | 38 | | 0 |
| Protein–RNA intermolecular | | 189 | |
| Total dihedral angle restraints | | | 11 |
| Protein | | | |
| $\phi$ | 0 | | |
| $\psi$ | 0 | | |
| Nucleic acid | | | |
| Base pair | | | 0 |
| Sugar pucker | | | 11 |
| Backbone | | | 0 |
| Based on A-form geometry | | | 0 |
| | | | |
| **Structure statistics** | | | |
| Violations (mean and s.d.) | | | |
| Number of distance constraints (>0.3 Å) (Å) | 5.4 | | |
| Dihedral angle constraints (º) | 0 | | |
| Max. dihedral angle violation (º) | 0 | | |
| Max. distance constraint violation (Å) | 0.58 | | |
| Deviations from idealized geometry | | | |
| Bond lengths (Å) | | | |
| Bond angles (º) | | | |
| Average pairwise r.m.s. deviation** (Å) | | | |
| Protein | | | |
| Heavy | 1.23 ± 0.24 | | |
| Backbone | 0.96 ± 0.27 | | |
| RNA | | | |
| All RNA heavy | | | 1.42 ± 0.41 |
| Complex | | | |
| Protein and RNA heavy | | | 1.15 ± 0.22 |

** Protein r.m.s. deviation was calculated using residues 126 to 278 for the ensemble of 10 refined structures. RNA r.m.s. deviation was calculated using nucleotides 105 to 112 for the ensemble of 10 refined structures.

## Supplementary Table 3: Sequencing and cross-link statistics

### Sequencing statistics

| sample name | Fl1 | Fl3 | Fl9 | Fl15 |
|---|---|---|---|---|
| after demultiplexing, adaper and quality trimming | 41961968 | 51648913 | 36102079 | 6603314 |
| uniquely mapping reads (STAR) | 15088434 | 15972767 | 8701315 | 3037369 |
| percentage of mapped reads | 35.96% | 30.93% | 24.10% | 46.00% |
| reads after deduplication | 791843 | 943385 | 1031678 | 41713 |
| percentage of uniquely mapped reads | 5.25% | 5.91% | 11.80% | 1.37% |

| sample name | RRM2 | RRM4 | RRM5 | RRM11 |
|---|---|---|---|---|
| after demultiplexing, adaper and quality trimming | 32641321 | 53631872 | 24151382 | 6063720 |
| uniquely mapping reads (STAR) | 8437501 | 15769120 | 10842843 | 1932186 |
| percentage of mapped reads | 25.85% | 29.40% | 44.90% | 31.86% |
| reads after deduplication | 307088 | 429353 | 71468 | 40080 |
| percentage of uniquely mapped reads | 3.64% | 2.72% | 0.66% | 2.07% |

| sample name | RS6 | RS7 | RS8 | negative |
|---|---|---|---|---|
| after demultiplexing, adaper and quality trimming | 29899294 | 51284284 | 1042128 | 8193366 |
| uniquely mapping reads (STAR) | 10772426 | 17937572 | 245601 | 4145409 |
| percentage of mapped reads | 36.03% | 34.98% | 23.57% | 50.59% |
| reads after deduplication | 410404 | 367486 | 11557 | 35023 |
| percentage of uniquely mapped reads | 3.81% | 2.05% | 4.71% | 0.84% |

### Cross-link statistics

| sample name | FL (Fl1, Fl3, Fl9) |
|---|---|
| size of merged files | 2766906 |
| number of cross-link sites (= read starts) | 1404256 |
| number of significant cross-link sites | 301191 |
| number of cross-link clusters | 46000 |
| number of clusters that do not overlap with a cluster from the negative sample | 45052 |

| sample name | RRM (RRM2, RRM4, RRM5) |
|---|---|
| size of merged files | 807909 |
| number of cross-link sites (= read starts) | 454707 |
| number of significant cross-link sites | 79214 |
| number of cross-link clusters | 21662 |
| number of clusters that do not overlap with a cluster from the negative sample | 20801 |

| sample name | RS (RS6, RS7) | negative |
|---|---|---|
| size of merged files | 777890 | 35023 |
| number of cross-link sites (= read starts) | 423135 | 24321 |
| number of significant cross-link sites | 74188 | 2812 |
| number of cross-link clusters | 24719 | 1255 |
| number of clusters that do not overlap with a cluster from the negative sample | 23859 | |

Sequencing Statistics: Table with information about the number of reads in the individual samples (columns) after different steps of the analysis pipeline (rows). Number of reads after preprocessing; number of reads that could be mapped to a unique position in the reference genome using STAR (percentage of mapped reads out of all reads), number of mapped reads after removal of PCR duplicates (percentage of deduplicated reads out of all uniquely mapped reads). Cross-link Statistics: Table with information about the cross-link sites per merged sample (columns) at different steps of the iCount analysis pipeline (rows). Number of deduplicated reads in the merged sample; number of cross-link sites, that is the number of positions in the genome with at least one read start; number of significant cross-link site as computed by iCount; number of cross-link clusters (cross-link sites less than three nucleotides apart are merged into clusters); final number of clusters in each sample (all clusters that overlap a cluster from the negative sample are removed).
